# A cohort study of self-perception of ageing and all-cause mortality among older adults in China: a multiple mediators analysis

**DOI:** 10.1186/s12889-024-18895-y

**Published:** 2024-05-23

**Authors:** Junling Gao, Huashuai Chen, Hao Chen, Yingwei Chen, Jixiang Xu, Yujie Wang, Zhizhong Wang

**Affiliations:** 1 School of Public Health, Fudan University, Shanghai, 200032 China; 2 Collaborative Innovation Cooperative Unit of National Clinical Research Center for Geriatric Diseases, Shanghai, 200032 China; 3 Core unit of Shanghai Clinical Research Center for Geriatric Diseases, Shanghai, 200032 China; 4 Business School, Xiangtan University, Xiangtan, Hunan 411105 China; 5 Geriatrics Dept, First Dongguan Affiliated Hospital, Guangdong Medical University, Dongguan, 523808 China; 6 School of Public Health, Guangdong Medical University, Dongguan, 523808 China

**Keywords:** Self-perception of ageing, All-cause mortality, Mediation, Social participation

## Abstract

**Background:**

Positive self-perception of aging (SPA) is a well-known predictor of longevity, while how and to what extent SPA is linked with all-cause mortality among older adults is still unclear. This study aims to elucidate the relationship between positive SPA and all-cause mortality and its potential mediators among Chinese older adults.

**Methods:**

This is a 20-year dynamic cohort study conducted among 22,957 older adults aged ≥ 65 years old from a nationally representative sample. Positive SPA was measured using a validated 7-item scale. Potential mediators including health behaviors and social participation were collected using a self-reported questionnaire. Cox proportional hazards regression models were conducted to examine the association between positive SPA and all-cause mortality. A mediation analysis was conducted to determine whether health behaviors and social participation mediated the association between SPA and all-cause mortality.

**Results:**

Throughout follow-up (median [interquartile range], 46 [21–84] months), all-cause mortality was 87.4%. Compared with older adults with the lowest quartile positive SPA, hazard ratio(HR) of all-cause mortality among older adults with the second, third, and fourth quartile of positive SPA was 0.96(95%CI:0.93-1.00), 0.93(95%CI:0.90–0.99), and 0.92(95%CI:0.87–0.96) respectively after controlling for all potential mediators and covariates. The mediation analysis showed that regular daily vegetable intake, physical activity, and high social participation explained 41.1-48.5% of the variance in the association between positive SPA and all-cause mortality.

**Conclusions:**

In this cohort study, we found that high positive SPA was associated with decreased all-cause mortality directly, and indirectly through healthy lifestyle behaviors and social participation. These findings suggest that interventions targeted at promoting or maintaining positive SPA may contribute to healthy ageing among older adults in China.

**Supplementary Information:**

The online version contains supplementary material available at 10.1186/s12889-024-18895-y.

## Introduction

Life expectancy for adults aged ≥ 60 years has improved globally from 18.8 to 21.1% between the year 2000 and 2019 [1]. Prior studies have demonstrated that there are differences in health and longevity among older adults who demonstrate similar risk factors [2], which suggests that individual differences may impact longevity and health.

Self-perception of aging (SPA) refers to internalized older individuals’ beliefs about their own ageing [3]. Several studies have indicated that SPA is an independent determinant of mortality [3–8], and health [9–13] after controlling for other risk factors. According to stereotype embodiment theory (SET) [14] and self-perception theory [15], SPA can influence health outcomes through three possible aspects: psychosocial, behavioral, and physiological. Numerous studies have found that positive SPA was associated with more healthy behaviors [13, 16–20] including eating a balanced diet, increased physical activity, and screening or treatment compliance. Studies also have found that a positive SPA was associated with positive psychosocial traits, such as self-efficacy [11, 21], social participation [22], and control of life [23]. Finally, a recent study reported that individuals with a more positive SPA had lower levels of C-reactive protein (CRP), a biomarker of cumulative stress-related inflammation [13].

The existing evidence supports several possible explanations of how SPA is linked with longevity and health [6, 24, 25]. Further, SET suggests that SPA utilizes multiple pathways to influence longevity and health [14]. To our knowledge, no longitudinal study has examined how health behaviors and social participation simultaneously mediate the relationship between SPA and risk of all-cause mortality.

Mediation analysis allows proceeding from the question “Does it work?” to the question “How does it work?” [26]. Causal mediation analysis is considered for time-to-event outcomes and survival analysis model to explore the causal pathways of exposure-outcome [27], which decomposes the total effects of exposure (e.g., SPA) to the outcome (e.g., mortality) into direct association and indirect association (through mediators, e.g., health behaviors).

A previous study has found that more negative SPA was associated with higher mortality, which could be mediated by a healthy lifestyle among very old Chinese adults during the 8-year follow-up period [6]. However, there were few study to examine the association between positive SPA and mortality among whole old adults. To further clarify how SPA affects longevity, we examined the SPA’s direct association and indirect association through healthy behaviors and social participation to all-cause mortality among 22,957 Chinese older adults in a 20-year community-based cohort study.

## Method

### Study participants

Data for the present study was abstracted from the Chinese Longitudinal Healthy Longevity Study (CLHLS), which is an ongoing dynamic cohort study among community dwellings adults aged ≥ 65 years. The participants in CLHLS were randomly selected from 22 of China’s 31 provinces where the total population constituted about 85% of the total population in China in 2000 and 82% in 2010. Further details on sampling processes and quality control of data from the CLHLS have been published elsewhere [28]. Eight waves of data collection have been conducted: 1998, 2000, 2002, 2005, 2008/2009, 2011/2012, 2014, and 2018.

For this study, a total of 43,025 participants were enrolled separately in the years 1998, 2000, 2002, 2005, 2008, 2011, and 2014, and were followed up in 2018. Participants with any missing value for any variable were excluded to avoid a misclassification bias. Therefore, 22,957 participants were included in the present study (see Supplementary Fig. 1 in Additional File 1).

### Measurements

#### Positive SPA

The seven item positive SPA was abstracted from the Positive Affect and Negative Affect schedule scale [30] and the Attitude Toward Own Aging subscale of the Philadelphia Geriatric Center Morale Scale [31]. The seven items were: (1) Do you look on the bright side of things? (2) Do you keep things neat and clean? (3) Can you make your own decisions concerning your personal affairs? (4) Do you feel as happy as when you were young? (5) Do you feel fearful or anxious? (6) Do you feel lonely and isolated? (7) Do you feel useless? Participants rated each item using five response options: *always*, *often*, *sometimes*, *seldom*, *never*. The positive SPA scale has been validated by previous studies [32–34]. In this study, for the first four positive items, the response options were coded as *always* = 5; *often* = 4; *sometimes* = 3; *seldom* = 2; *never* = 1. For the latter three negative items, the response options were coded as *always* = 1; *often* = 2; *sometimes* = 3; seldom = 4; *never* = 5. The summed score of all items was used to define positive SPA, which was divided into quartiles for analysis where 1–16 was 1st quartile,17–19 was 2nd quartile, 20–22 was 3rd quartile, and 23–28 was 4th quartile. A higher quartile indicates a more positive SPA.

### Potential mediators

#### Health behaviors

Based on the literature [16, 35, 36], current smoking, drinking, physical activity, daily fresh fruit consumption, and vegetable intake were defined as health behaviors. Smoking, drinking, and physical activity were assessed by asking participants the following items “Do you smoke/drink/exercise or not at present,” which were each answered on a yes/no scale. Those who responded *yes* to each of the questions were considered to engage in that behavior.

Daily fresh fruit intake and vegetable intake were assessed by asking the following question: “How often do you eat fresh fruit/vegetables,” which was answered using the following scale: *almost every day, except winter, occasionally*, and *rarely or never*. Those who answered *almost every day* were coded as *regularly*, while those who answered *except winter, occasionally, rarely or never* were coded as *irregular*.

#### Social participation

Social participation was assessed by asking participants how often they engaged in the following 8 social activities presently [34]: (1) housework (e.g., cooking and caring for children), (2) growing vegetables & engaging in other fieldwork, (3) engaging in garden work, (4) reading newspapers/books, (5) raising domestic animals/pets, (6) playing cards/mahjong, (7) watching TV or listening to the radio, and (8) attending religious activities. The participants enrolled in the cohort in 1998 and 2000, responded to each question: *almost every day* (coded as 2), *sometimes* (coded as 1), and *never* (coded as 0). The participants enrolled in the cohort in 2002, 2005, 2008, 2011, and 2014, responded to each question: *almost every day* (coded as 4), *not every day but at least once in a week* (coded as 3), *not every week but once in a month* (coded as 2), not every month but sometimes (coded as 1), and *never* (coded as 0). A total social participation score was created by summing the responses of all 8 social activities when participants were newly enrolled in the cohort separately. Given the different response scales, we harmonized social participation into high and low using a median split of the scores to increase the comparability of measures completed before 2002, and those completed after 2002.

#### Covariates

Besides, age (5-year categories), sex (male, female), years of schooling (coded as 0, 1–6, and ≥ 7 years), marital status (married, unmarried [including widowed, divorced, and never-married]), self-rated health (codes as *good*, *general*, and *poor*), we also included cognitive function, functional capacity, and number of noncommunicable chronic diseases comorbidity (NCDs) as covariates. Methods used to measure cognitive function, functional capacity and number of NCDs can be found in Supplementary Table 1 in Additional File 1.

#### Mortality

Information on death was collected from the next-of-kin [37]. Duration of follow-up was calculated by subtracting the time interval at the first interview date from the date of death. Survivors at the wave after which they were last surveyed were censored at the time of the survey.

#### Statistical analysis

The proportion was calculated for categorical variables in each category sub-stratified by positive SPA. Multivariable Cox proportional hazards regression models were used to estimate the association between positive SPA and all-cause mortality. The crude and adjusted models were used to examine the association between positive SPA and mortality without and with adjusting for any covariates or potential mediators.

**Fig. 1:**
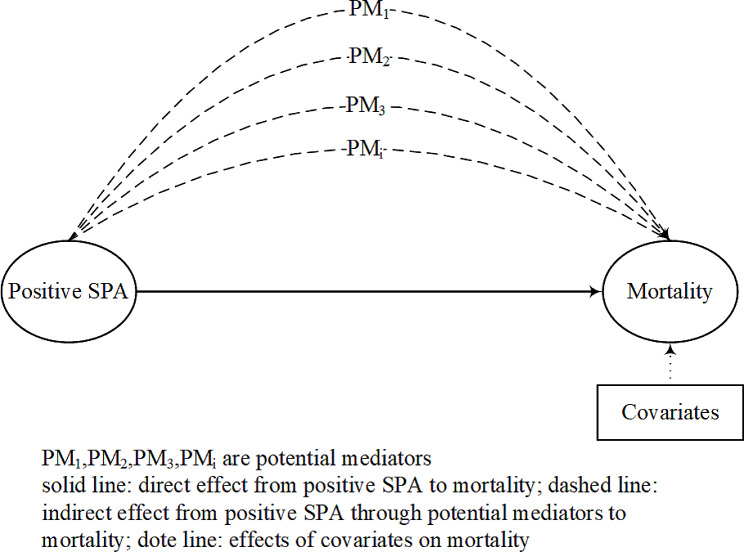
A framework for multiple mediators between positive self-perception of ageing and mortality

To determine whether health behaviors and social participation mediated the association between positive SPA and mortality after adjusting for covariates, we used the general multiple third-variable effect analysis method (TVEA) proposed by Yu et al [38, 39], which allows consideration of multiple mediators simultaneously and the use of linear and non-linear predictive models for estimating mediation association (Fig. 1). The Bootstrap method was used to estimate the variances and confidence intervals. Specifically, bias-corrected 95% confidence intervals (95% CI) were estimated by repeating the analysis on 1000 bootstrapped samples.

Statistical analyses were completed using R 4.1.2 [40] using the *survival* [41] and *mmabig* [42] packages. The *mmabig* package identifies a mediator as a variable that is significantly correlated with the predictor, and outcome, given that all other related factors are included in the model. In this study, if a potential mediator was not associated with mortality, it was removed from the mediation analysis. All statistical tests were 2-sided and considered significant at *P* < .05.

In sensitivity analyses for the mediation analysis, we first refitted the model by repeating the analysis on 250 and 500 bootstrapped samples. Second, we ran the model excluding the participants with a follow-up duration of less than 6 months to avoid reverse causation. Finally, because the oldest-old adults may not provide accurate information, we performed the model among participants aged < 100 years old.

## Results

### Baseline characteristics of participants

Baseline characteristics of included versus excluded participants are presented in Supplementary Table 2 in Additional File 1. Except for cognitive function, all other characteristics were not different among participants who were included and excluded from the present study.

Table 1 shows the baseline characteristics of all included participants, 44.1% were men, 9.7% were < 70 years old 31.1% were ≥ 95 years old, 64.4% were illiterate, and 29.0% were married. Over half of the participants (55.9%) reported their health as *good*, 58.8% reported no NCDs; 17.8% demonstrated abnormal cognitive function and 23.4% reported *disabled* functional capacity at baseline. The prevalence of current smoking and drinking was 19.8% and 22.3%, respectively. The proportion of regular daily intake of fruits/vegetables, and engaging in physical activity was 28.2%, 84.5%, and 28.1%, respectively. The proportion of high social participation was 47.5%. The proportions of the first, second, third, and fourth quartiles of positive SPA were 28.6%, 26.1%, 26.6%, and 18.7%, respectively. Participants with higher positive SPA also had higher levels of social participation(Table 1).

**Table 1:** Baseline characteristics of participants in CLHLS (1998–2018)

		Total, No. (%) (*N* = 22,957)	Positive SPA, No. (%)				*P*-value
			1st quartile (*N* = 6,562)	2nd quartile (*N* = 5,984)	3rd quartile (*N* = 6,111)	4th quartile (*N* = 4,300)	
Death	No	2892 (12.6)	543 (8.3)	687 (11.5)	830 (13.6)	832 (19.3)	< 0.001
	Yes	20,065 (87.4)	6019 (91.7)	5297 (88.5)	5281 (86.4)	3468 (80.7)	
Sex	Male	10,120 (44.1)	2407 (36.7)	2573 (43.0)	2876 (47.1)	2264 (52.7)	< 0.001
	Female	12,837 (55.9)	4155 (63.3)	3411 (57.0)	3235 (52.9)	2036 (47.3)	
Age	65-	2231 (9.7)	364 (5.5)	527 (8.8)	633 (10.4)	707 (16.4)	< 0.001
	70-	1327 (5.8)	265 (4.0)	348 (5.8)	354 (5.8)	360 (8.4)	
	75-	1387 (6.0)	319 (4.9)	401 (6.7)	346 (5.7)	321 (7.5)	
	80-	3356 (14.6)	883 (13.5)	827 (13.8)	975 (16.0)	671 (15.6)	
	85-	3253 (14.2)	949 (14.5)	860 (14.4)	846 (13.8)	598 (13.9)	
	90-	4267 (18.6)	1295 (19.7)	1170 (19.6)	1122 (18.4)	680 (15.8)	
	95-	7136 (31.1)	2487 (37.9)	1851 (30.9)	1835 (30.0)	963 (22.4)	
Years of schooling	0	14,786 (64.4)	4892 (74.6)	3955 (66.1)	3738 (61.2)	2201 (51.2)	< 0.001
	1–6	5229 (22.8)	1214 (18.5)	1384 (23.1)	1502 (24.6)	1129 (26.3)	
	7-	2942 (12.8)	456 (6.9)	645 (10.8)	871 (14.3)	970 (22.6)	
Marriage status	Unmarried	16,289 (71.0)	5234 (79.8)	4337 (72.5)	4163 (68.1)	2555 (59.4)	< 0.001
	Married	6668 (29.0)	1328 (20.2)	1647 (27.5)	1948 (31.9)	1745 (40.6)	
Self-rated health	Good	12,837 (55.9)	2303 (35.1)	3300 (55.1)	4011 (65.6)	3223 (75.0)	< 0.001
	General	7590 (33.1)	2873 (43.8)	2102 (35.1)	1712 (28.0)	903 (21.0)	
	Bad	2530 (11.0)	1386 (21.1)	582 (9.7)	388 (6.3)	174 (4.0)	
Cognitive function	Normal	18,877 (82.2)	4617 (70.4)	4939 (82.5)	5313 (86.9)	4008 (93.2)	< 0.001
	Abnormal	4080 (17.8)	1945 (29.6)	1045 (17.5)	798 (13.1)	292 (6.8)	
Functional capacity	Active	17,578 (76.6)	4534 (69.1)	4595 (76.8)	4806 (78.6)	3643 (84.7)	< 0.001
	Disabled	5379 (23.4)	2028 (30.9)	1389 (23.2)	1305 (21.4)	657 (15.3)	
Number of NCDs	0	13,495 (58.8)	3600 (54.9)	3571 (59.7)	3746 (61.3)	2578 (60.0)	< 0.001
	1	6543 (28.5)	1987 (30.3)	1675 (28.0)	1694 (27.7)	1187 (27.6)	
	2	2919 (12.7)	975 (14.9)	738 (12.3)	671 (11.0)	535 (12.4)	
Daily fruit intake	Irregular	16,480 (71.8)	5397 (82.2)	4422 (73.9)	4225 (69.1)	2436 (56.7)	< 0.001
	Regular	6477 (28.2)	1165 (17.8)	1562 (26.1)	1886 (30.9)	1864 (43.3)	
Daily vegetable intake	Irregular	3562 (15.5)	1458 (22.2)	935 (15.6)	800 (13.1)	369 (8.6)	< 0.001
	Regular	19,395 (84.5)	5104 (77.8)	5049 (84.4)	5311 (86.9)	3931 (91.4)	
Current smoking	Yes	4547 (19.8)	1007 (15.3)	1188 (19.9)	1302 (21.3)	1050 (24.4)	< 0.001
	No	18,410 (80.2)	5555 (84.7)	4796 (80.1)	4809 (78.7)	3250 (75.6)	
Drinking	Yes	5126 (22.3)	1007 (15.3)	1188 (19.9)	1302 (21.3)	1050 (24.4)	< 0.001
	No	17,831 (77.7)	5555 (84.7)	4796 (80.1)	4809 (78.7)	3250 (75.6)	
Physical activity	Yes	6442 (28.1)	1167 (17.8)	1264 (21.1)	1544 (25.3)	1151 (26.8)	< 0.001
	No	16,515 (71.9)	5395 (82.2)	4720 (78.9)	4567 (74.7)	3149 (73.2)	
Social Participation	Low	12,043 (52.5)	4395 (67.0)	3238 (54.1)	2924 (47.8)	1486 (34.6)	< 0.001
	High	10,914 (47.5)	2167 (33.0)	2746 (45.9)	3187 (52.2)	2814 (65.4)	

### Positive SPA and all-cause mortality

The median duration of follow-up for mortality ascertainment was 46 months (range: 0.1–252 months; interquartile range: 21–84 months), during which 20,065 (87.4%) deaths from all causes were identified (Table 1). All-cause mortality among participants within the first, second, third, and fourth quartiles of positive SPA were 91.7%, 88.5%, 86.4%, and 80.7%, respectively (Table 1). Cox proportional hazards regression models showed that compared to participants with the lowest quartile of positive SPA, the hazard ratios (HR) for all-cause mortality among participants with the second, third, and fourth quartile of positive SPA were 0.80 (95% CI: 0.77–0.83), 0.75 (95% CI: 0.72–0.78) and 0.60 (95% CI: 0.57–0.63) without controlling for any covariates and potential mediators (**Crude Model** in Table 2). After controlling for all covariates and potential mediators, the HRs of all-cause mortality among older adults with higher quartiles of positive SPA were lower than that of older adults within the lowest quartile of positive SPA (**Adjusted Model** in Table 2). However, all the hazard ratios in the **Adjusted Model** than their counterparts in the **Crude Model**, indicates that other factors may mediate the association between positive SPA and all-cause mortality.

**Table 2:** Associations of positive SPA with all-cause mortality in Cox models

Characteristic	Crude Model		Adjusted Model	
	HR(95% CI)	*p*-value	HR(95% CI)	*p*-value
**Positive SPA**				
1st quartile	-		-	
2nd quartile	0.80(0.77–0.83)	< 0.001	0.96(0.93-1.00)	0.060
3rd quartile	0.75(0.72–0.78)	< 0.001	0.93(0.90–0.99)	0.033
4th quartile	0.60(0.57–0.63)	< 0.001	0.92(0.87–0.96)	< 0.001
**Gender** (female vs. male)			0.79(0.76–0.82)	< 0.001
**Age**				
65-			-	
70-			1.73(1.57–1.91)	< 0.001
75-			2.65(2.42–2.90)	< 0.001
80-			4.26(3.94–4.62)	< 0.001
85-			5.47(5.05–5.94)	< 0.001
90-			7.29(6.72–7.91)	< 0.001
95-			9.67(8.91–10.50)	< 0.001
**Years of schooling**				
0			-	
1–6			1.05(1.01–1.09)	0.025
7-			1.03(0.97–1.08)	0.317
**Marriage status (**married vs. unmarried)			0.84(0.81–0.87)	< 0.001
**Self-rated health**				
Good			-	
General			1.07(1.04–1.11)	< 0.001
Bad			1.21(1.15–1.27)	< 0.001
**Cognitive function (**abnormal vs. normal)			1.20(1.15–1.25)	< 0.001
**Functional capacity (**disabled vs. active)			1.44(1.39–1.49)	< 0.001
**Number of NCDs**				
0			-	
1			1.06(1.03–1.09)	< 0.001
2			1.11(1.06–1.16)	< 0.001
**Fruit intake (**regular vs. irregular)			0.98(0.96-1.00)	0.275
**Vegetable intake (**regular vs. irregular)			0.93(0.91–0.96)	< 0.001
**Current smoking** (No vs. Yes)			0.95(0.89–1.01)	0.088
**Current drinking** (No vs. Yes)			1.02(0.99–1.04)	0.334
**Physical activity** (Yes vs. No)			0.95(0.93–0.98)	< 0.001
**Social participation** (high vs. low)			0.82(0.79–0.85)	< 0.001

### Mediators of the association between positive SPA and all-cause mortality

After controlling for all covariates, compared with participants with the lowest quartile of positive SPA, the mediation analysis found the following (Table 3).

**Table 3:** Estimated direct and indirect effect sizes of SPA With All-Cause Mortality through mediators in CLHLS (1998–2018)

	Effect estimation		HR(95%CI)	%Mediated, mean(95%CI)
	Effect Sizes, mean(95%CI)	*P*-value		
**2nd quartile of positive SPA**				
**Direct effect**	-0.041(-0.074 - -0.007)	0.016	0.960(0.929–0.993)	51.5(24.1–78.9)
**Indirect effect through**	-0.034(-0.040 - -0.028)	< 0.001	0.967(0.961–0.972)	48.5(21.1–75.9)
Daily vegetable intake (Regular)	-0.004(-0.006- -0.002)	< 0.001	0.996(0.994–0.998)	5.2(1.1–9.3)
Physical activity (Yes)	-0.005(-0.007 - -0.002)	< 0.001	0.995(0.993–0.998)	6.0(1.5–11.9)
Social participation (High)	-0.026(-0.030 - -0.021)	< 0.001	0.974(0.970–0.979)	36.6(16.1–57.2)
**Total effect**	-0.075(-0.109 - -0.041)	< 0.001	0.928(0.897–0.960)	
**3rd quartile of positive SPA**				
**Direct effect**	-0.076(-0.112 - -0.040)	< 0.001	0.927(0.894–0.961)	57.9(45.2–70.7)
**Indirect effect through**	-0.053(-0.061 - -0.046)	< 0.001	0.948(0.941–0.955)	42.1(29.3–54.8)
Daily vegetable intake (Regular)	-0.006(-0.008 - -0.003)	< 0.001	0.994(0.992–0.997)	4.4(1.9–6.9)
Physical activity (Yes)	-0.009(-0.013 - -0.005)	< 0.001	0.991(0.987–0.995)	7.1(3.2–11.0)
Social participation (High)	-0.039(-0.044- -0.033)	< 0.001	0.962(0.957–0.968)	30.5(21.2–39.9)
**Total effect**	-0.134(-0.166 - -0.093)	< 0.001	0.879(0.847–0.911)	
**4th quartile of positive SPA**				
**Direct effect**	-0.123(-0.161 - -0.084)	< 0.001	0.884(0.851–0.919)	58.9(50.0-67.7)
**Indirect effect through**	-0.084(-0.096 - -0.073)	< 0.001	0.919(0.908–0.929)	41.1(32.3–50.0)
Daily vegetable intake (Regular)	-0.008(-0.012 - -0.004)	< 0.001	0.992(0.988–0.996)	3.9(1.9–5.9)
Physical activity (Yes)	-0.015(-0.023 - -0.008)	< 0.001	0.985(0.977–0.992)	7.5(3.7–11.3)
Social participation (High)	-0.061(-0.069 - -0.053)	< 0.001	0.941(0.933–0.948)	29.7(23.2–36.2)
**Total effect**	-0.207(-0.246–0.169)	< 0.001	0.813(0.782–0.845)	

First, older adults within the second quartile of positive SPA had a lower risk of death (HR: 0.928, 95% CI: 0.897–0.960). The total indirect effects through three mediators(regular daily vegetable intake, physical activity, and high social participation) were − 0.034 (95% CI: −0.040–−0.028). The relative effect suggests that the direct effect explained 51.5% (95% CI: 24.1–78.9%) of the total variance of associations between all-cause mortality among older adults with the lowest and second quartile of positive SPA, and the remaining 48.5% (95% CI: 21.1–75.9%) of the total variance was explained by regular daily vegetable intake (5.2%, 95% CI: 1.1–9.3%), physical activity (6.0%, 95% CI: 1.5–11.9%) and high social participation (36.6%, 95% CI: 16.1–57.2%).

Second, older adults within the third quartile of positive SPA had a lower risk of death (HR: 0.879, 95% CI: 0.847–0.911). The total indirect effect through these three mediators was − 0.053 (95% CI: −0.061–−0.046). The relative effect suggests that the direct effect explained 57.9% (95% CI: 45.2–70.7%) of the total variance of associations between all-cause mortality among older adults within the lowest and the third quartiles of positive SPA, and the remaining 42.1% (95% CI: 29.3–54.8%) of the total variance was explained by regular daily vegetable intake (4.4%, 95% CI: 1.9–6.9%), physical activity (7.1%, 95% CI: 3.2–11.0%) and high social participation (30.5%, 95% CI: 21.2–39.9%).

Third, older adults within the fourth quartile of positive SPA had a lower risk of death (HR: 0.813, 95% CI: 0.782–0.845). The total indirect effect through these three mediators was − 0.084 (95% CI: −0.096–−0.073). The relative effect suggests that the direct effect explained 58.9% (95% CI: 50.0–67.7%) of the total variance of associations between all-cause mortality among older adults within the lowest quartile and third quartile for positive SPA, and the remaining 41.1% (95% CI: 32.3–50.0%) of the total variance was explained by regular daily vegetable intake (3.9%, 95% CI: 1.9–5.9%), physical activity (7.5%, 95% CI: 3.7–11.3%) and high social participation (29.7%, 95% CI: 23.2–36.2%).

**Fig. 2:**
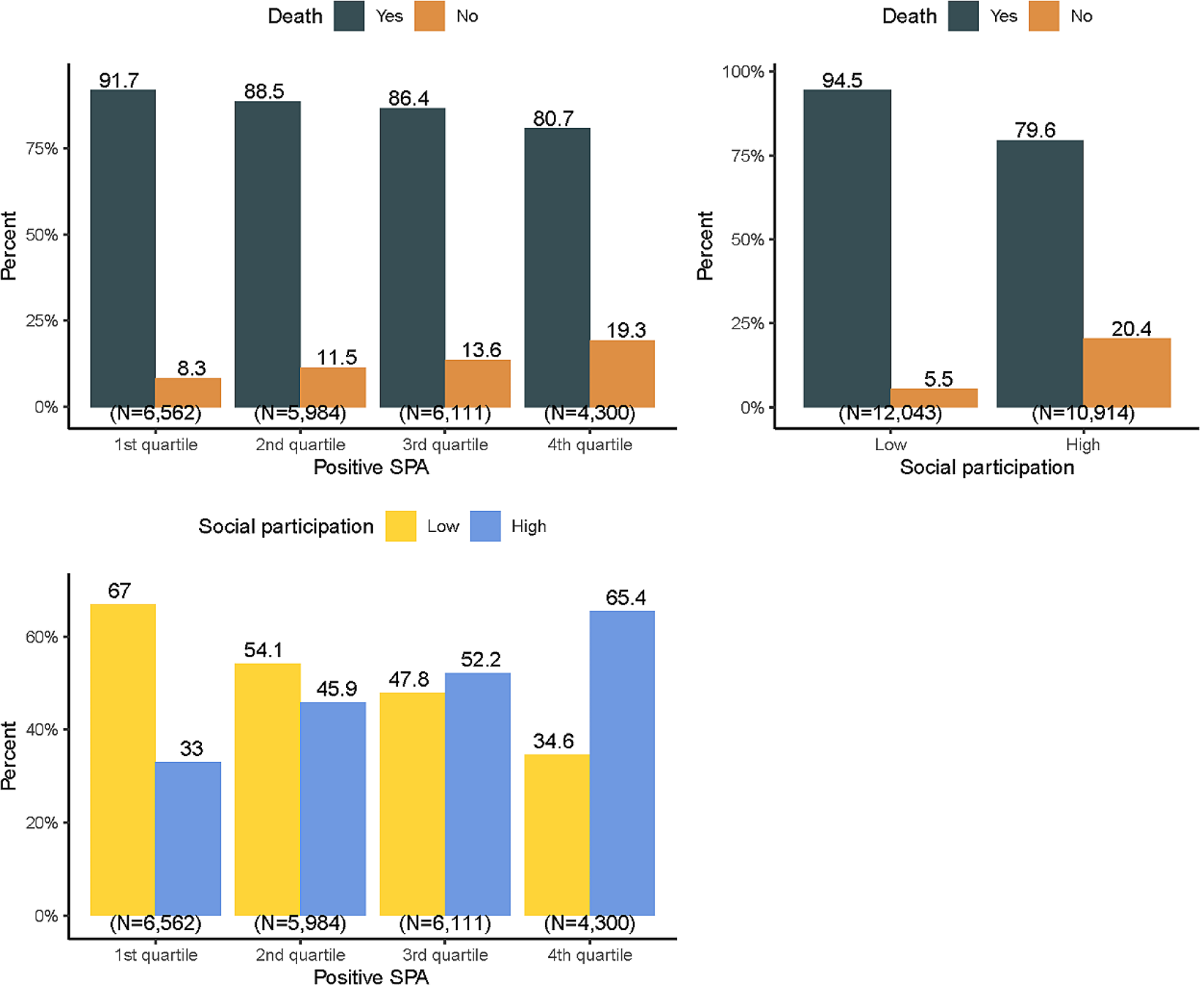
Mediation of social participation between positive SPA and all-cause mortality

Figure 2 shows that all-cause mortality among participants with high social participation was lower than that among participants with low social participation (94.5% vs. 79.6%) (upper right panel), while the proportion of participants with high social participation was greater with greater positive SPA (lower left panel). All-cause mortality was decreased with increased positive SPA (upper left panel). Therefore, accounting for social participation may increase the gap in hazard rates between older adults with higher positive SPA and lower positive SPA. Results showing mediation results for regular daily vegetable intake and physical activity are presented in Supplementary Fig. 2 and Fig. 3 in Additional File 1.

### Sensitivity analyses

Four different sensitivity analyses showed there were no significantly differences between the results of main analysis and sensitivity analysis (see Supplementary Tables 3–6 in Additional File 1).

## Discussion

In this large, nationally representative prospective cohort study of older adults in China, we found that a more positive SPA was associated with a lower risk of all-cause mortality. This finding is consistent with a study from Western countries that found a more positive SPA is associated with greater longevity [13]. On other side, two studies in China found that more negative SPA [6] and self-perceived uselessness [33] were associated with increased all-cause mortality. According to stereotype embodiment theory (SET) [14], self-perception of aging may be unconsciously internalized across the life course. A study also found that holding negative stereotypes about aging earlier in life was associated with worse health among older individuals [43]. These findings suggest that broadly promoting positive SPA across the life course may contribute to healthy aging.

To the best of our knowledge, this is the first study to examine whether health behaviors and social participation simultaneously mediate the association between positive SPA and all-cause mortality among Chinese older adults. From a behavioral pathway, previous studies have found that a positive SPA was positively associated with preventive health behaviors that have been found to have close relationship with longevity [16, 18]. More positive SPA was consistently associated with better self-rated health, less obesity, better performance of the activities of daily living, and better cognitive functioning revealed by a systematic review that pooled twenty-one longitudinal studies [19]. Specifically, a recent study found that preventive health behaviors mediated the association between negative SPA and all-cause mortality [6]. In this study, we also found that higher SPA may decrease all-cause mortality through regular daily vegetable intake and physical activity. From the psychosocial pathway, we found that higher positive SPA promoted social participation, which in turn decreased all-cause mortality among older adults. These findings partly support the stereotype embodiment theory, which hypothesizes that SPA utilizes multiple pathways to influence health and longevity [14]. Our findings also support social participation as a protective factor for mortality [44–46] and suggest that individual motivations may be significant facilitators of social participation [47]. Despite the importance of healthy lifestyle [36, 48] and social participation [49] in pursuing healthy aging, only a small proportion of older adults practice them [48, 50]. Our findings have important practical implications that provide evidence for interventions aim to promote or maintain high positive SPA, which may lead to older adults practicing healthy lifestyle behaviors and engaging in social participation.

### Strengths and limitations

A major strength of our study includes the estimation of multiple potential pathways from positive SPA to mortality through healthy lifestyle behaviors and social participation in a 20-year prospective cohort study with a large, nationally representative sample of older adults in China. However, our study also has several limitations. First, a higher proportion of loss to follow-up and excluding participants with any missing data to avoid a misclassification bias, and unmeasured or residual confounders may distort the present findings. Second, the SPA, healthy lifestyle behaviors, and social participation were all measured at the same time point of baseline, thus against causal inference in our mediation model that high social participation mediates the effects of SPA on the risk of all-cause mortality; besides, participants’ SPA, health behaviors, and social participation may have changed during follow-up [4], we are failed to capture longitudinal changes in SPA, healthy lifestyle behaviors, and social participation that may impact mortality. Third, the mediation analysis was based on the assumption that there was no confounding between the variables of interest in the present study [51]. Finally, there may be unmeasured variables that explain the relationship between SPA and mortality that were not measured.

## Conclusions

In conclusion, this study found that a more positive SPA is associated with a lower risk of all-cause mortality, and this association is partly mediated by healthy lifestyle behaviors and social participation. Those findings provide new evidence for stereotype embodiment theory that claims SPA utilizes multiple pathways to influence longevity and health. And suggest that interventions that aim to promote and maintain high positive SPA may contribute to healthy aging among older adults in China.

### Electronic supplementary material

Below is the link to the electronic supplementary material.

Supplementary Material 1

## Data Availability

The CLHLS datasets are publicly available at the Peking University of Healthy Aging and Development( http://chads.nsd.pku.edu.cn). Researchers may obtain the datasets after sending a data user agreement to the CLHLS team.

## Abbreviations

CLHLS: Chinese Longitudinal Healthy Longevity Study
CRP: C-Reactive Protein
NCDs: Noncommunicable Chronic Diseases Comorbidity
SET: Stereotype Embodiment Theory
SPA: Positive self-perception of aging
STROBE: Strengthening the Reporting of Observational Studies in Epidemiology
TVEA: Third-Variable Effect Analysis Method
