# Supplementary material for: A cohort study of self-perception of ageing and all-cause mortality among older adults in China: a multiple mediators analysis
Source: BMC Public Health. 2024 May 23;24:1382. doi: 10.1186/s12889-024-18895-y (PMC11112801; doi:10.1186/s12889-024-18895-y)
Supplement: Supplementary file 1 — Supplementary Material 1 [file 12889_2024_18895_MOESM1_ESM.docx]

**Supplementary** **Appendix**

Supplementary Figure 1: Flowchart of participants from 1998-2018 in CLHLS

Supplementary Figure 2: mediation of daily vegetable intake between positive SPA and all-cause mortality

Supplementary Figure 3: mediation of physical activity between positive SPA and all-cause mortality

Supplementary Table 1: Methods to measure cognitive function, functional capacity and number of NCDs

Supplementary Table 2: baseline characteristics of included and excluded participants, No. (%)

Supplementary Table 3: Estimated direct and indirect effect sizes of SPA With All-Cause Mortality through mediators in CLHLS (1998-2018) (Bootstrapped samples n=250)

Supplementary Table 4: Estimated direct and indirect effect sizes of SPA With All-Cause Mortality through mediators in CLHLS (1998-2018) (Bootstrapped samples n=500)

Supplementary Table 5: Estimated direct and indirect effect sizes of SPA With All-Cause Mortality through mediators among participants with duration of follow-up ≥ 6 months in CLHLS (1998-2018)

Supplementary Table 6: Estimated direct and indirect effect sizes of SPA With All-Cause Mortality through mediators among participants aged<100 years old in CLHLS (1998-2018)

Total: 43,025, lost:12,218, excluded with missing data: 8,030, Analysis in the current study:22,957

**1998**: 9,093

Lost: 894

Died: 3,368

Followed survivors: 4,831

Newly: 6,214

**2000**: 11,045

Lost: 1,541

Died: 3,307

Followed survivors: 6,224

Newly: 9,590

**2002**: 15,814

Lost: 1,983

Died: 5,800

Followed survivors: 8,031

Newly: 7,067

**2005**: 15,098

Lost: 2,862

Died: 5,061

Followed survivors: 7,175

Newly:8,840

**2008**: 16,015

Lost: 2,737

Died: 5,388

Followed survivors: 7,890

Newly: 1,276

**2011**: 9,166

Lost: 782

Died: 2,879

Followed survivors: 5,669

Newly: 1,125

**2014**: 6,794

Lost: 1,446

Died: 2,092

**2018**, survivors:3,256

**Supplementary Figure 1. Flowchart of participants from 1998-2018 in CLHLS**


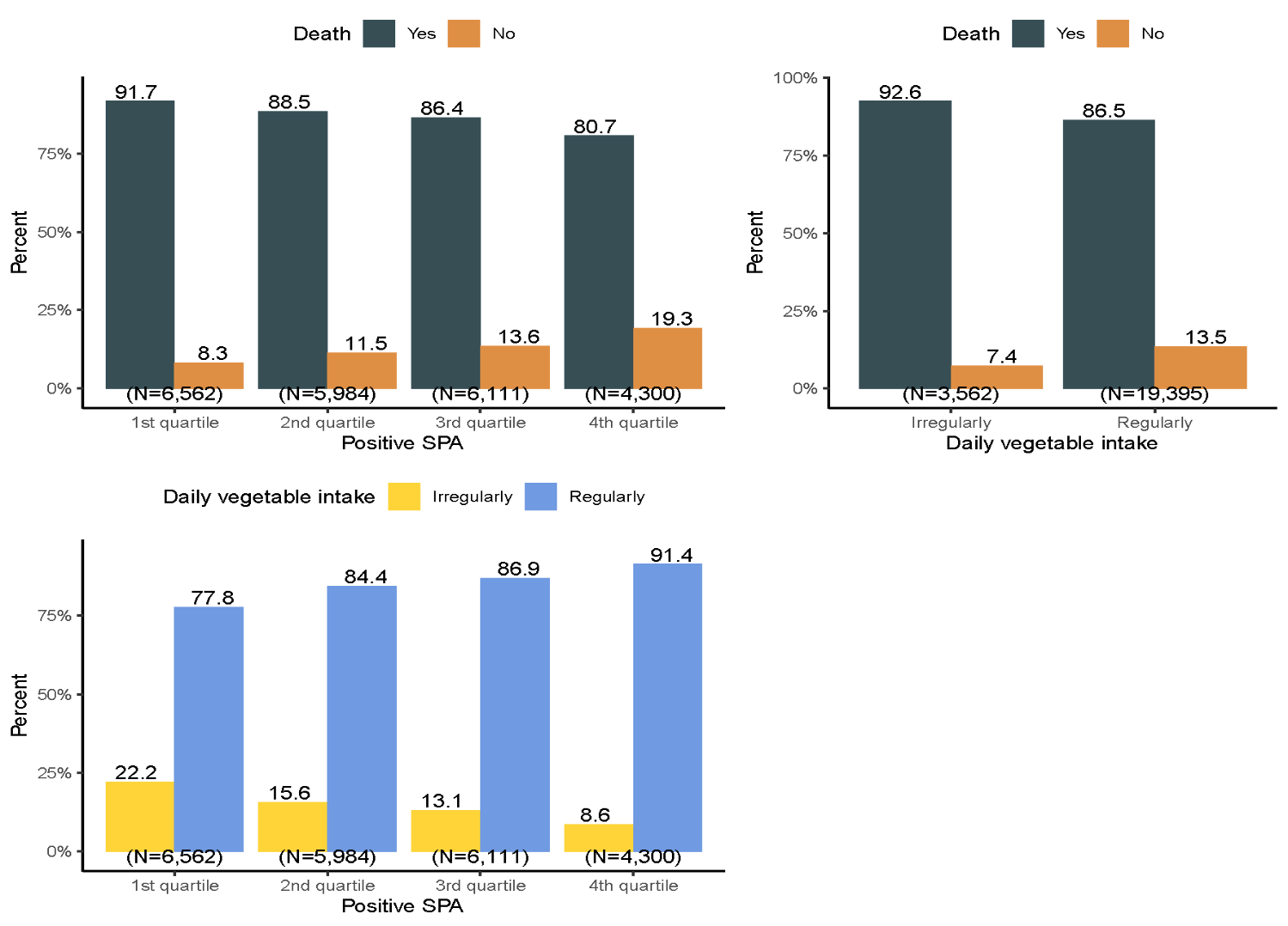


**Supplementary Figure 2. mediation of daily vegetable intake between positive SPA and all-cause mortality**


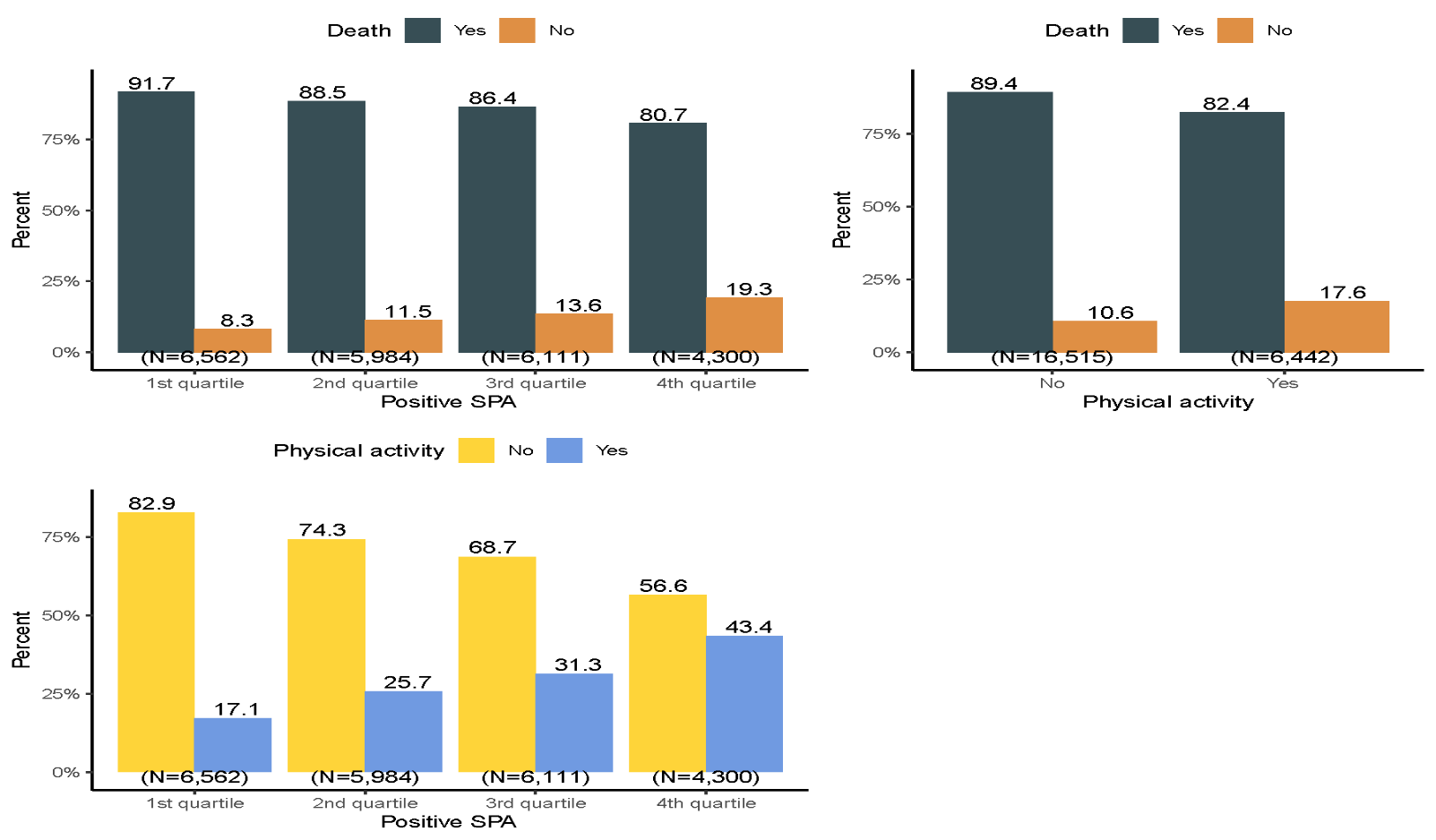


**Supplementary Figure 3 mediation of physical activity between positive SPA and all-cause mortality**

**Supplementary Table 1. Methods to measure cognitive function, functional capacity and number of NCDs**

| 1. Cognitive function was assessed using the Mini Mental State Examination-Modified Chinese version (MMSE-MC) with scores ranging from 0 to 30^1^. The MMSE-MC is a screening measure that assesses orientation, retention, attention and calculation, recall, and language and verbal fluency. Participants with scores ≥19 were classified as having normal cognitive function, while participants with scores <19 were classified as having abnormal cognitive function37. |
| --- |
| **2. Functional capacity^2^** was assessed by asking participants whether they have difficulties in six activities of daily living (ADLs): (1) bathing, (2) dressing, (3) toileting, (4) indoor transfer, (5) continence, and (6) feeding. Participants were classified as active if they need no assistance in any ADL. If participants required assistance in any area, they were classified as disabled. |
| **3. number of NCDs**: Participants were asked to answer the question “Are you suffering from any of the following diagnosed chronic diseases,” which included: (1) hypertension, (2) diabetes, (3) heart disease, (4) stroke, cerebrovascular disease, (5) bronchitis, pulmonary emphysema, asthma, pneumonia, (6) pulmonary tuberculosis, (7) cataract, (8) glaucoma, (9) cancer, (10) prostate tumor, (11) gastric or duodenal ulcer, (12) Parkinson’s disease. If participants reported zero diseases, NCD was coded as 0; if they reported having one disease, NCD was coded as 1, and if they reported having two or more diseases, NCD was coded as ≥2. |

**Reference**

1. Gao MY, Yang M, Kuang WH, Qiu PY. Factors and validity analysis of Mini-Mental State Examination in Chinese elderly people[in Chinese]. Beijing Da Xue Xue Bao Yi Xue Ban. Jun 18 2015;47(3):443-9.

2. Yi Z, Vaupel JW. Functional Capacity and Self–Evaluation of Health and Life of Oldest Old in China. Journal of Social Issues. 2002;58(4):733-748. doi:https://doi.org/10.1111/1540-4560.00287

| **Supplementary Table 2. baseline characteristics of included and excluded participants, No. (%)** | | | | |
| --- | --- | --- | --- | --- |
|  | | **Included participants**  **(N=22,957)** | **Excluded participants**  **(N=8,030)** | **P** |
| Sex | Male | 10120 (44.1) | 3501 (43.6) | .453 |
|  | Female | 12837 (55.9) | 4529 (56.4) |  |
| Age | 65- | 2231 (9.7) | 763 (9.5) | .400 |
|  | 70- | 1327 (5.8) | 458 (5.7) |  |
|  | 75- | 1387 (6.0) | 482 (6.0) |  |
|  | 80- | 3356 (14.6) | 1122 (14.0) |  |
|  | 85- | 3253 (14.2) | 1108 (13.8) |  |
|  | 90- | 4267 (18.6) | 1492 (18.6) |  |
|  | 95- | 7136 (31.1) | 2606 (32.4) |  |
| Years of schooling^a^ | 0 | 14786 (64.4) | 5081 (65.0) | .551 |
|  | 1-6 | 5229 (22.8) | 1735 (22.2) |  |
|  | 7- | 2942 (12.8) | 1001 (12.8) |  |
| Marriage status^b^ | Unmarried | 16289 (71.0) | 5725 (71.5) | .362 |
|  | Married | 6668 (29.0) | 2283 (28.5) |  |
| Self-rated health^c^ | Good | 12837 (55.9) | 1842 (39.1) | .085 |
|  | General | 7590 (33.1) | 1749 (37.1) |  |
|  | Bad | 2530 (11.0) | 1118 (23.7) |  |
| Cognitive function^d^ | Normal | 18877 (82.2) | 3252 (80.9) | .042 |
|  | Abnormal | 4080 (17.8) | 768 (19.1) |  |
| Functional capacity | Active | 17578 (76.6) | 6143 (76.5) | .901 |
|  | Disabled | 5379 (23.4) | 1887 (23.5) |  |
| Number of NCDs | 0 | 13495 (58.8) | 4519 (58.8) | .954 |
|  | 1 | 6543 (28.5) | 2299 (28.6) |  |
|  | 2 | 2919 (12.7) | 1012 (15.1) |  |
| Daily fruit intake**^e^** | Irregular | 16480 (71.8) | 5819 (72.6) | .148 |
|  | Regular | 6477 (28.2) | 2193 (27.4) |  |
| Daily vegetable intake^f^ | Irregular | 3562 (15.5) | 1271 (15.9) | .440 |
|  | Regular | 19395 (84.5) | 6733 (84.1) |  |
| Current smoking^g^ | Yes | 4547 (19.8) | 1610 (20.1) | .601 |
|  | No | 18410 (80.2) | 6409 (79.9) |  |
| Drinking^h^ | Yes | 5126 (22.3) | 1756 (21.9) | .473 |
|  | No | 17831 (77.7) | 6247 (78.1) |  |
| Physical activity^k^ | No | 6442 (28.1) | 2265 (28.4) | .565 |
|  | Yes | 16515 (71.9) | 5711 (71.6) |  |
| Social Participation^i^ | Low | 12043 (52.5) | 4143 (51.7) | .260 |
|  | High | 10914 (47.5) | 3866 (48.3) |  |
| Positive SPA^l^ | 1^st^ quartile | 6562(28.6) | 1432(29.8) | .103 |
|  | 2^nd^ quartile | 5984(26.1) | 1278(26.6) |  |
|  | 3^rd^ quartile | 6111(26.6) | 1264(26.3) |  |
|  | 4^th^ quartile | 4300(18.7) | 838(17.4) |  |

Missing number among excluded participants: a=213, b=22, c=3321, d=4010, e=18**, f=26, g=11, h=27, k=54, i=21, l=3218

**Supplementary Table 3 Estimated direct and indirect effect sizes of SPA With All-Cause Mortality through mediators in CLHLS (1998-2018) (Bootstrapped samples n=250)**

|  | **Effect estimation** | | **HR(95%CI)** | **%Mediated, mean(95%CI)** |
| --- | --- | --- | --- | --- |
|  | **Effect Sizes, mean(95%CI)** | **P-value** |  |  |
| **2^nd^ quartile of positive SPA** |  |  |  |  |
| **Direct effect** | -0.041(-0.076 - -0.007) | .020 | 0.960(0.927-0.993) | 52.1(25.4-78.7) |
| **Indirect effect through** | -0.034(-0.039 - -0.028) | <.001 | 0.967(0.962-0.972) | 47.9(21.3-74.6) |
| Daily vegetable intake (Regular) | -0.004(-0.006 - -0.002) | <.001 | 0.996(0.994-0.998) | 5.2(0.9-9.4) |
| Physical activity (Yes) | -0.005(-0.007 - -0.002) | <.001 | 0.995(0.993-0.998) | 6.7(1.4-12.1) |
| Social participation (High) | -0.026(-0.030 - -0.021) | <.001 | 0.974(0.970-0.979) | 36.2(16.3-56.2) |
| **Total effect** | -0.075(-0.110 - -0.040) | <.001 | 0.928(0.895-0.961) |  |
| **3^rd^ quartile of positive SPA** |  |  |  |  |
| **Direct effect** | -0.076(-0.112 - -0.040) | <.001 | 0.927(0.894-0.961) | 58.0(44.6-71.4) |
| **Indirect effect through** | -0.053(-0.060 - -0.046) | <.001 | 0.948(0.942-0.955) | 42.0(28.6-55.4) |
| Daily vegetable intake (Regular) | -0.006(-0.008 - -0.003) | <.001 | 0.994(0.992- 0.997) | 4.4(1.8-7.1) |
| Physical activity (Yes) | -0.009(-0.014 - -0.004) | <.001 | 0.991(0.986-0.996) | 7.1(2.8-11.4) |
| Social participation (High) | -0.039(-0.044 - -0.033) | <.001 | 0.962(0.957-0.968) | 30.5(20.7-40.2) |
| **Total effect** | -0.129(-0.165 - -0.093) | <.001 | 0.879(0.848-0.911) |  |
| **4^th^ quartile of positive SPA** |  |  |  |  |
| **Direct effect** | -0.123(-0.161 - -0.085) | <.001 | 0.884(0.851-0.919) | 59.0(50.4-67.6) |
| **Indirect effect through** | -0.084(-0.096 - -0.073) | <.001 | 0.919(0.908-0.930) | 41.0(32.4-49.6) |
| Daily vegetable intake (Regular) | -0.008(-0.012 - -0.004) | <.001 | 0.992(0.988-0.996) | 3.9(1.9-6.0) |
| Physical activity (Yes) | -0.015(-0.023 - -0.007) | <.001 | 0.985(0.977-0.993) | 7.4(3.3-11.6) |
| Social participation (High) | -0.061(-0.069 - -0.052) | <.001 | 0.941(0.933-0.949) | 29.5(23.2-35.8) |
| **Total effect** | -0.208(-0.246 - -0.169) | <.001 | 0.812(0.782-0.845) |  |

**Supplementary Table 4 Estimated direct and indirect effect sizes of SPA With All-Cause Mortality through mediators in CLHLS (1998-2018) (Bootstrapped samples n=500)**

|  | **Effect estimation** | | **HR(95%CI)** | **%Mediated, mean(95%CI)** |
| --- | --- | --- | --- | --- |
|  | **Effect Sizes, mean(95%CI)** | **P-value** |  |  |
| **2^nd^ quartile of positive SPA** |  |  |  |  |
| **Direct effect** | -0.041(-0.075 - -0.007) | .018 | 0.960(0.928-0.993) | 51.4(25.3-77.6) |
| **Indirect effect through** | -0.035(-0.040 - -0.029) | <.001 | 0.966(0.961-0.971) | 48.6(22.4-74.7) |
| Daily vegetable intake (Regular) | -0.004(-0.006 - -0.002) | <.001 | 0.996(0.994-0.998) | 5.2(1.3-9.1) |
| Physical activity (Yes) | -0.005(-0.007 - -0.002) | <.001 | 0.995(0.993-0.998) | 6.7(1.7-11.7) |
| Social participation (High) | -0.026(-0.031 - -0.022) | <.001 | 0.974(0.969-0.978) | 36.7(13.7-61.1) |
| **Total effect** | -0.075(-0.109 - -0.042) | <.001 | 0.928(0.897-0.959) |  |
| **3^rd^ quartile of positive SPA** |  |  |  |  |
| **Direct effect** | -0.075(-0.109 - -0.042) | <.001 | 0.928(0.897-0.959) | 57.7(45.2-70.1) |
| **Indirect effect through** | -0.054(-0.061 - -0.046) | <.001 | 0.947(0.941-0.955) | 42.3(29.9-54.8) |
| Daily vegetable intake (Regular) | -0.006(-0.008 - -0.003) | <.001 | 0.994(0.992- 0.997) | 4.4(2.0-6.9) |
| Physical activity (Yes) | -0.009(-0.014 - -0.004) | <.001 | 0.991(0.986-0.996) | 7.1(3.1-11.2) |
| Social participation (High) | -0.039(-0.045 - -0.033) | <.001 | 0.962(0.956-0.968) | 30.8(21.6-40.0) |
| **Total effect** | -0.129(-0.163 - -0.095) | <.001 | 0.879(0.850-0.909) |  |
| **4^th^ quartile of positive SPA** |  |  |  |  |
| **Direct effect** | -0.123(-0.161 - -0.084) | <.001 | 0.884(0.851-0.919) | 58.7(50.1-67.2) |
| **Indirect effect through** | -0.085(-0.096 - -0.074) | <.001 | 0.919(0.908-0.929) | 41.3(32.8-49.9) |
| Daily vegetable intake (Regular) | -0.008(-0.012 - -0.004) | <.001 | 0.992(0.988-0.996) | 3.9(1.9-6.0) |
| Physical activity (Yes) | -0.015(-0.023 - -0.008) | <.001 | 0.985(0.977-0.992) | 7.5(3.5-11.5) |
| Social participation (High) | -0.062(-0.070 - -0.053) | <.001 | 0.940(0.932-0.948) | 29.9(23.6-36.1) |
| **Total effect** | -0.208(-0.247 - -0.169) | <.001 | 0.812(0.781-0.845) |  |

**Supplementary Table 5 Estimated direct and indirect effect sizes of SPA With All-Cause Mortality through mediators among participants with duration of follow-up ≥ 6 months in CLHLS (1998-2018)**

|  | **Effect estimation** | | **HR(95%CI)** | **%Mediated, mean(95%CI)** |
| --- | --- | --- | --- | --- |
|  | **Effect Sizes, mean(95%CI)** | **P-value** |  |  |
| **2^nd^ quartile of positive SPA** |  |  |  |  |
| **Direct effect** | -0.035(-0.068 - -0.002) | .038 | 0.966(0.934-0.998) | 48.2(13.6-82.8) |
| **Indirect effect through** | -0.032(-0.038 - -0.027) | <.001 | 0.969(0.963-0.973) | 51.8(17.2-86.4) |
| Daily vegetable intake (Regular) | -0.003(-0.005- -0.001) | .003 | 0.997(0.995-0.999) | 5.2(0.8-9.2) |
| Physical activity (Yes) | -0.004(-0.006 - -0.001) | .002 | 0.996(0.995- 0.999) | 6.0(0.2- 11.7) |
| Social participation (High) | -0.025(-0.030 - -0.021) | <.001 | 0.975(0.970- 0.979) | 40.7(13.2-68.1) |
| **Total effect** | -0.067(-0.101- -0.034) | <.001 | 0.935(0.904-0.967) |  |
| **3^rd^ quartile of positive SPA** |  |  |  |  |
| **Direct effect** | -0.072(-0.107 - -0.033) | <.001 | 0.931(0.899-0.964) | 58.0(44.1-71.8) |
| **Indirect effect through** | -0.050(-0.058 - -0.042) | <.001 | 0.951(0.944-0.959) | 42.0(28.2-55.9) |
| Daily vegetable intake (Regular) | -0.005(-0.008 - -0.002) | .001 | 0.995(0.992-0.998) | 4.2(1.5-6.9) |
| Physical activity (Yes) | -0.007(-0.012 - -0.002) | .006 | 0.993(0.988-0.998) | 5.9(1.6-10.2) |
| Social participation (High) | -0.038(-0.044 - -0.032) | <.001 | 0.963(0.957-0.969) | 31.9(21.3-42.5) |
| **Total effect** | -0.122(-0.157 - -0.087) | <.001 | 0.885(0.855-0.917) |  |
| **4^th^ quartile of positive SPA** |  |  |  |  |
| **Direct effect** | -0.112(-0.153 - -0.071) | <.001 | 0.894(0.858- 0.931) | 58.1(47.9-68.3) |
| **Indirect effect through** | -0.079(-0.068- -0.091) | <.001 | 0.924(0.913-0.934) | 41.9(31.7-52.5) |
| Daily vegetable intake (Regular) | -0.007(-0.011- -0.003) | .001 | 0.993(0.989-0.997) | 3.8(1.6-6.1) |
| Physical activity (Yes) | -0.012(-0.020- -0.004) | .003 | 0.988(0.989-0.996) | 6.3(2.0-10.6) |
| Social participation (High) | -0.060(-0.068- -0.052) | <.001 | 0.942(0.934-0.949) | 31.8(23.8-39.7) |
| **Total effect** | -0.192(-0.233- -0.150) | <.001 | 0.825(0.792-0.861) |  |

**Supplementary Table 6 Estimated direct and indirect effect sizes of SPA With All-Cause Mortality through mediators among participants aged<100 years old in CLHLS (1998-2018)**

|  | **Effect estimation** | | **HR(95%CI)** | **%Mediated, mean(95%CI)** |
| --- | --- | --- | --- | --- |
|  | **Effect Sizes, mean(95%CI)** | **P-value** |  |  |
| **2^nd^ quartile of positive SPA** |  |  |  |  |
| **Direct effect** | -0.036(-0.069 - -0.003) | .033 | 0.965(0.933-0.997) | 48.2(13.6-82.8) |
| **Indirect effect through** | -0.032(-0.037 - -0.027) | <.001 | 0.969(0.964-0.973) | 51.8(17.2-86.4) |
| Daily vegetable intake (Regular) | -0.003(-0.005- -0.001) | .003 | 0.997(0.995-0.999) | 5.2(0.8-9.2) |
| Physical activity (Yes) | -0.004(-0.006 - -0.001) | .002 | 0.996(0.995- 0.999) | 6.0(0.2- 11.7) |
| Social participation (High) | -0.025(-0.029 - -0.021) | <.001 | 0.975(0.971- 0.979) | 40.7(13.2-68.1) |
| **Total effect** | -0.068(-0.101- -0.035) | <.001 | 0.934(0.904-0.966) |  |
| **3^rd^ quartile of positive SPA** |  |  |  |  |
| **Direct effect** | -0.072(-0.109 - -0.036) | <.001 | 0.931(0.897-0.965) | 58.3(44.2-72.4) |
| **Indirect effect through** | -0.050(-0.058 - -0.042) | <.001 | 0.951(0.944-0.959) | 41.7(27.6-55.8) |
| Daily vegetable intake (Regular) | -0.005(-0.008 - -0.002) | .001 | 0.995(0.992-0.998) | 4.1(1.4-6.8) |
| Physical activity (Yes) | -0.007(-0.012 - -0.002) | .006 | 0.993(0.988-0.998) | 5.9(1.7-10.1) |
| Social participation (High) | -0.038(-0.044 - -0.032) | <.001 | 0.963(0.957-0.969) | 31.7(20.5-42.9) |
| **Total effect** | -0.122(-0.109 - -0.039) | <.001 | 0.885(0.897-0.962) |  |
| **4^th^ quartile of positive SPA** |  |  |  |  |
| **Direct effect** | -0.112(-0.153 - -0.071) | <.001 | 0.894(0.858- 0.931) | 58.8(49.6-68.1) |
| **Indirect effect through** | -0.079(-0.068- -0.091) | <.001 | 0.924(0.913-0.934) | 41.2(31.9-50.4) |
| Daily vegetable intake (Regular) | -0.007(-0.011- -0.003) | .001 | 0.993(0.989-0.997) | 3.7(1.6-5.8) |
| Physical activity (Yes) | -0.012(-0.020- -0.004) | .003 | 0.988(0.989-0.996) | 6.3(2.0-10.5) |
| Social participation (High) | -0.060(-0.068- -0.052) | <.001 | 0.942(0.934-0.949) | 31.2(23.9-38.5) |
| **Total effect** | -0.192(-0.233- -0.150) | <.001 | 0.825(0.792-0.861) |  |
